# Real‐world efficacy and safety of universal 8‐week glecaprevir/pibrentasvir in patients with chronic hepatitis C with early chronic kidney disease or pre‐end‐stage renal disease: Insights from a nationwide hepatisis C virus registry in Taiwan

**DOI:** 10.1002/kjm2.12929

**Published:** 2025-01-19

**Authors:** Szu‐Jen Wang, Chung‐Feng Huang, Te‐Sheng Chang, Ching‐Chu Lo, Chao‐Hung Hung, Chien‐Wei Huang, Lee‐Won Chong, Pin‐Nan Cheng, Ming‐Lun Yeh, Cheng‐Yuan Peng, Chien‐Yu Cheng, Jee‐Fu Huang, Ming‐Jong Bair, Chih‐Lang Lin, Chi‐Chieh Yang, Hsing‐Tao Kuo, Tsai‐Yuan Hsieh, Tzong‐Hsi Lee, Pei‐Lun Lee, Wen‐Chih Wu, Chih‐Lin Lin, Wei‐Wen Su, Sheng‐Shun Yang, Chia‐Chi Wang, Jui‐Ting Hu, Lein‐Ray Mo, Chun‐Ting Chen, Yi‐Hsiang Huang, Chun‐Chao Chang, Chia‐Sheng Huang, Guei‐Ying Chen, Chien‐Neng Kao, Chi‐Ming Tai, Chun‐Jen Liu, Mei‐Hsuan Lee, Pei‐Chien Tsai, Chia‐Yen Dai, Jia‐Horng Kao, Han‐Chieh Lin, Wang‐Long Chuang, Kuo‐Chih Tseng, Chi‐Yi Chen, Shu‐Chi Wang, Ming‐Lung Yu

**Affiliations:** ^1^ Graduate Institute of Clinical Medicine Kaohsiung Medical University Kaohsiung Taiwan; ^2^ Division of Hepatogastroenterology, Department of Internal Medicine Shin Huey Shin Hospital Kaohsiung Taiwan; ^3^ Division of Gastroenterology, Department of Internal Medicine Yuan's General Hospital Kaohsiung Taiwan; ^4^ Hepatobiliary Division, Department of Internal Medicine and Hepatitis Center Kaohsiung Medical University Hospital, Kaohsiung Medical University Kaohsiung Taiwan; ^5^ Ph.D. Program in Translational Medicine, College of Medicine Kaohsiung Medical University, Academia Sinica Kaohsiung Taiwan; ^6^ Division of Hepatogastroenterology, Department of Internal Medicine ChiaYi Chang Gung Memorial Hospital Chiayi Taiwan; ^7^ College of Medicine Chang Gung University Taoyuan Taiwan; ^8^ Division of Gastroenterology, Department of Internal Medicine St. Martin De Porres Hospital Chiayi Taiwan; ^9^ Division of Hepatogastroenterology, Department of Internal Medicine Kaohsiung Chang Gung Memorial Hospital Kaohsiung Taiwan; ^10^ Division of Gastroenterology Kaohsiung Armed Forces General Hospital Kaohsiung Taiwan; ^11^ Division of Hepatology and Gastroenterology, Department of Internal Medicine Shin Kong Wu Ho‐Su Memorial Hospital Taipei Taiwan; ^12^ School of Medicine Fu‐Jen Catholic University New Taipei City Taiwan; ^13^ Division of Gastroenterology and Hepatology, Department of Internal Medicine National Cheng Kung University Hospital, College of Medicine, National Cheng Kung University Tainan Taiwan; ^14^ Hepatitis Research Center, College of Medicine; Center for Liquid Biopsy and Cohort Research Kaohsiung Medical University Kaohsiung Taiwan; ^15^ Centers for Digestive Medicine, Department of Internal Medicine China Medical University Hospital Taichung Taiwan; ^16^ School of Medicine China Medical University Taichung Taiwan; ^17^ Division of Infectious Diseases, Department of Internal Medicine, Taoyuan General Hospital Ministry of Health and Welfare Taoyuan Taiwan; ^18^ School of Medicine and Doctoral Program of Clinical and Experimental Medicine, College of Medicine and Center of Excellence for Metabolic Associated Fatty Liver Disease National Sun Yat‐sen University Kaohsiung Taiwan; ^19^ Division of Gastroenterology, Department of Internal Medicine Taitung Mackay Memorial Hospital Taitung Taiwan; ^20^ Mackay Medical College New Taipei City Taiwan; ^21^ Liver Research Unit, Department of Hepato Gastroenterology and Community Medicine Research Center, Chang Gung Memorial Hospital at Keelung, College of Medicine Chang Gung University Keelung Taiwan; ^22^ Department of Gastroenterology, Division of Internal Medicine Show Chwan Memorial Hospital Changhua Taiwan; ^23^ Division of Gastroenterology and Hepatology, Department of Internal Medicine Chi Mei Medical Center Yongkang District Tainan Taiwan; ^24^ Division of Gastroenterology, Department of Internal Medicine Tri Service General Hospital, National Defense Medical Center Taipei Taiwan; ^25^ Division of Gastroenterology and Hepatology Far Eastern Memorial Hospital New Taipei City Taiwan; ^26^ Division of Gastroenterology and Hepatology, Department of Internal Medicine Chi Mei Medical Center Tainan Taiwan; ^27^ Wen‐Chih Wu Clinic, Fengshan Kaohsiung Taiwan; ^28^ Department of Gastroenterology, Renai Branch Taipei City Hospital Taipei Taiwan; ^29^ Department of Gastroenterology and Hepatology Changhua Christian Hospital Changhua Taiwan; ^30^ Division of Gastroenterology and Hepatology, Department of Internal Medicine Taichung Veterans General Hospital Taichung Taiwan; ^31^ Taipei Tzu Chi Hospital, Buddhist Tzu Chi Medical Foundation and School of Medicine Tzu Chi University Taipei Taiwan; ^32^ Liver Center Cathay General Hospital Taipei Taiwan; ^33^ Division of Gastroenterology Tainan Municipal Hospital (Managed By Show Chwan Medical Care Corporation) Tainan Taiwan; ^34^ Division of Gastroenterology, Department of Internal Medicine Tri Service General Hospital Penghu Branch National Defense Medical Center Taipei Taiwan; ^35^ Division of Gastroenterology and Hepatology, Department of Medicine Taipei Veterans General Hospital Taipei Taiwan; ^36^ Institute of Clinical Medicine, School of Medicine National Yang‐Ming Chiao Tung University Taipei Taiwan; ^37^ Division of Gastroenterology and Hepatology, Department of Internal Medicine Taipei Medical University Hospital Taipei Taiwan; ^38^ Division of Gastroenterology and Hepatology, Department of Internal Medicine, School of Medicine, College of Medicine Taipei Medical University Taipei Taiwan; ^39^ Yang Ming Hospital Chiayi Taiwan; ^40^ Penghu Hospital Ministry of Health and Welfare Taiwan; ^41^ National Taiwan University Hospital Hsin‐Chu Branch Hsinchu Taiwan; ^42^ Division of Gastroenterology and Hepatology, Department of Internal Medicine, E‐Da Hospital I‐Shou University Kaohsiung Taiwan; ^43^ School of Medicine for International Students, College of Medicine I‐Shou University Kaohsiung Taiwan; ^44^ Hepatitis Research Center and Department of Internal Medicine National Taiwan University Hospital Taipei Taiwan; ^45^ Institute of Clinical Medicine National Yang‐Ming Chiao Tung University Taipei Taiwan; ^46^ School of Medicine Tzuchi University Hualien Taiwan; ^47^ Department of Internal Medicine, Dalin Tzu Chi Hospital Buddhist Tzu Chi Medical Foundation Chiayi Taiwan; ^48^ Division of Gastroenterology and Hepatology, Department of Medicine Ditmanson Medical Foundation Chiayi Christian Hospital Chiayi Taiwan; ^49^ Department of Medical Laboratory Science and Biotechnology Kaohsiung Medical University Kaohsiung Taiwan

**Keywords:** chronic hepatitis C (CHC), chronic kidney disease (CKD), direct‐acting antivirals (DAA), end‐stage renal disease (ESRD), real‐world

## Abstract

An 8‐week regimen of glecaprevir/pibrentasvir is recommended for treatment‐naïve patients with chronic hepatitis C (CHC). In alignment with the Taiwanese government's objective to eliminate hepatitis C by 2025, this study aimed to provide real‐world evidence on the use of this regimen in treatment‐naïve patients with chronic kidney disease (CKD) by using data from the Taiwan Association for the Study of the Liver HCV Registry (TACR). CKD was defined by an estimated glomerular filtration rate (eGFR) of <60 mL/min/1.73 m^2^ or higher with proteinuria persisting for over 3 months. Patients were categorized as having early CKD (eGFR ≥45 mL/min/1.73 m^2^) or pre‐end‐stage renal disease (pre‐ESRD) (eGFR <45 mL/min/1.73 m^2^). Among 1072 patients who received at least one dose of the regimen, 1054 had available data for assessing sustained virologic response at 12 weeks posttreatment (SVR12). The overall SVR12 rate was 99.6%, with rates of 99.7% for pre‐ESRD patients and 99.6% for early CKD patients. Subgroup analysis showed 100% efficacy for genotype 3 and dyslipidemia, 99.5% for diabetes, 99.4% for cardiovascular disease, 96.9% for a history of cerebral vascular accident, and 95.5% for patients with a history of drug injection or HIV co‐infection. Adverse events were reported in 16.8% of patients, with 0.8% experiencing serious events, and only two cases were treatment‐related. Renal function significantly improved, with overall eGFR increasing from 39.2 to 41.9 mL/min/1.73 m^2^. Early CKD patients showed an eGFR rise from 53.5 to 57.1, while pre‐ESRD patients improved from 27.1 to 29.2 at SVR12. The study concluded that the 8‐week regimen is highly effective, well‐tolerated, and associated with significant renal function improvement in treatment‐naïve CHC patients with both early CKD and pre‐ESRD.

## 1 INTRODUCTION

The hepatitis C virus (HCV) was first identified in 1989, and it has since become a crucial public health concern. On January 1, 2020, Polaris models estimated a global prevalence of 0.7% for HCV RNA‐positive individuals, with this corresponding to 56.8 million infections.^1^ Since 2016, significant strides have been made globally in managing hepatitis C virus (HCV). The World Health Organization (WHO) has set a goal to control the spread of the hepatitis C virus (HCV) by 2030. The WHO has expressed support for expanding HCV treatment by promoting decentralized testing, task shifting, and simplification of therapy with direct‐acting antivirals (DAAs) at the primary care level.^2^

Chronic HCV infection frequently advances to liver fibrosis and cirrhosis, and in cases involving bridging fibrosis or cirrhosis, it can eventually lead to hepatocellular carcinoma (HCC).^3^ Moreover, nearly two‐thirds of patients with chronic HCV infection (CHC) experience extrahepatic manifestations. These complications arise either from HCV infecting B lymphocytes, which can lead to conditions such as mixed cryoglobulinemia and non‐Hodgkin B‐cell lymphoma, or from chronic inflammation, which increases the risk of cardiovascular events such as stroke and coronary artery disease as well as kidney diseases and metabolic disorders.^4^

In addition to liver complications, HCV can lead to renal concerns such as glomerulopathies and tubulointerstitial damage, which leads to a high prevalence of chronic kidney disease (CKD).^5^ The relationship between CKD and HCV infection is reciprocal, with HCV serving as both a cause and a consequence of CKD.^6^ A systematic review and meta‐analysis of 15 longitudinal studies involving 2,299,134 patients in total revealed a significant association between positive anti‐HCV serologic status and an increased incidence of CKD.^6^ HCV infection is an independent risk factor for accelerated CKD progression and is associated with adverse outcomes in patients with end‐stage renal disease (ESRD), including higher rates of hepatic‐related hospitalizations, increased mortality, and reduced health‐related quality of life.^7^

Glecaprevir/pibrentasvir (GLE/PIB) is currently recommended as the standard treatment for CHC. In two clinical trials involving patients with CKD stages 3b–5, namely EXPEDITION‐4 and EXPEDITION‐5, the sustained virologic response (SVR) rates were reported to be 98% (102/104; 95% CI, 95%–100%) and 97% (98/101; 95% CI, 91.6%–99%), respectively, according to intention‐to‐treat (ITT) analysis. No serious AEs were identified that were attributable to the study drugs, and no grade 4 laboratory abnormalities were reported.^8^ Both the American Association for the Study of Liver Diseases/Infectious Diseases Society of America (AASLD/IDSA) and the European Association for the Study of the Liver (EASL) guidelines recommend a fixed‐dose combination of glecaprevir and pibrentasvir as the treatment of choice for patients with CHC and CKD.^9^, ^10^, ^11^

Achieving an SVR through hepatitis C antiviral therapy can help reduce both hepatic and extrahepatic complications, including cardiovascular and metabolic concerns.^12^ DAA can be employed as a faster, more effective, and safer treatment option. New pan‐genotypic DAAs, such as GLE/PIB, were reported to have high efficacy in an 8‐week regimen across all genotypes.^13^ However, real‐world data on their impact on extrahepatic manifestations, particularly regarding changes in estimated glomerular filtration rate (eGFR) and proteinuria in patients with CHC and CKD are limited.

The Taiwan Association for the Study of the Liver HCV Registry (TACR) adopted an 8–16‐week regimen of GLE/PIB, with the regimen based on cirrhosis status, treatment history, and HCV genotype, as outlined in the 2020 guidelines.^14^ Briefly, from February 2018 to March 2020, 16‐week GLE/PIB was approved for interferon‐experienced HCV genotype 3 patients. Eight‐week GLE/PIB was approved for treatment‐naïve and interferon‐experienced non‐cirrhotic patients, while 12‐week GLE/PIB was for treatment‐naïve and interferon‐experienced compensated‐cirrhotic patients. Since April 2020, 8‐week GLE/PIB has also been approved for treatment‐naïve patients with compensated liver cirrhosis. After updates to these guidelines were published by the US Food and Drug Administration, we provided real‐world evidence supporting an 8‐week GLE/PIB regimen for treatment‐naïve patients with cirrhosis, which was published in 2021.^15^ In line with the WHO's new treatment simplification policy, in the current study, we broadened our scope of research to include a wider range of patient profiles and viral characteristics.

This study assessed the real‐world efficacy, tolerability, and impact of an 8‐week regimen of glecaprevir/pibrentasvir (GLE/PIB) on renal function in patients with CHC and either early CKD or pre‐ESRD. This study employed data from the largest HCV registry in the world, employing the most extensive cohort of patients with CHC to date.

## 2 METHODS

### 2.1 Participants

Participants were identified from the TACR, a prospective, observational, nationwide cohort managed by the Taiwan Association for the Study of the Liver (TASL), which includes 53 medical institutes. This registry specifically targets patients with CHC treated with DAAs.^14^, ^15^, ^16^, ^17^, ^18^, ^19^ As of May 31, 2024, the TACR had registered a total of 1733 patients with CHC with CKD who had received GLE/PIB treatment. The current analysis focused on DAA treatment‐naïve patients aged >18 years who had received at least one dose of GLE/PIB. Patients undergoing hemodialysis or those lacking SVR12 data at the end of follow‐up were excluded from the study. The study protocol was approved by the Institutional Review Boards of the participating hospitals and adhered to the International Conference on Harmonization Guidelines for Good Clinical Practice. All patients provided written informed consent prior to enrollment in the registry.

The primary objective was to assess the achievement of a sustained virologic response at 12 weeks (SVR12), which was defined as maintaining undetectable HCV RNA levels (<12 or <25 IU/mL, depending on the laboratory) throughout a 12‐week posttreatment follow‐up period.

CKD was defined as an eGFR of <60 mL/min/1.73 m^2^ or an eGFR of >60 mL/min/1.73 m^2^ with proteinuria persisting for more than 3 months.^14^, ^15^ Early CKD and pre‐ESRD represent different stages of CKD. Early CKD includes stages 1 to 3a, which are characterized by an eGFR ranging from 45 to 60 mL/min/1.73 m^2^, regardless of the presence of proteinuria. Pre‐ESRD includes stages 3b to 5: Stage 3b is characterized by an eGFR of 30–44.9 mL/min/1.73 m^2^, stage 4 by an eGFR of 15–29.9 mL/min/1.73 m^2^, and stage 5 by an eGFR of <15 mL/min/1.73 m^2^, indicating a need for dialysis.^20^ The patients were categorized into two groups: those with an eGFR of ≥45 mL/min/1.73 m^2^ were classified as the early CKD group, and those with an eGFR of <45 mL/min/1.73 m^2^ were classified as the pre‐ESRD group.

Health‐care resource utilization was assessed by counting the number of clinic visits from the initiation of GLE/PIB treatment until the SVR12 survey visit. Drug adherence was measured by the ratio of actual doses taken to the expected 8‐week regimen for each patient throughout the treatment period. Changes in laboratory parameters were evaluated for patients both before treatment and at SVR12. Additionally, four comorbidities of interest were recorded from clinical charts, and the correlations between changes in eGFR, proteinuria, and these comorbidities were analyzed.

### 2.2 Statistical analysis

Group frequencies were compared using *χ* ^2^ tests with Yates' correction or Fisher's exact test as appropriate. Group means were compared using analysis of variance, Student's *t* test, or the Mann–Whitney *U* test and are presented as means and standard deviations. Paired *t* tests were employed to evaluate changes in laboratory data before and after DAA therapy. The efficacy of GLE/PIB was evaluated in both the ITT population, which included all patients who received at least one dose, and the modified intention‐to‐treat (mITT) population, defined as those who received at least one dose and had HCV RNA data available at week 12 posttreatment. Safety assessments included the identification of adverse events (AEs), serious adverse events (SAEs), and laboratory abnormalities in the ITT population. The eGFR was calculated using the Modification of Diet in Renal Disease equation,^16^ and changes in eGFR from baseline to week 12 posttreatment were assessed in the mITT population. The Fibrosis‐4 (FIB‐4) score was calculated using the formula: age (years) × aspartate transaminase (AST) [U/L]/(platelets [109/L] × (alanine transaminase [ALT] [U/L])1/2). All statistical analyses were conducted using SPSS 14.0 statistical software (SPSS, Chicago, IL, USA).

## 3 RESULTS

### 3.1 Baseline patient demographics and characteristics

**FIGURE 1:**
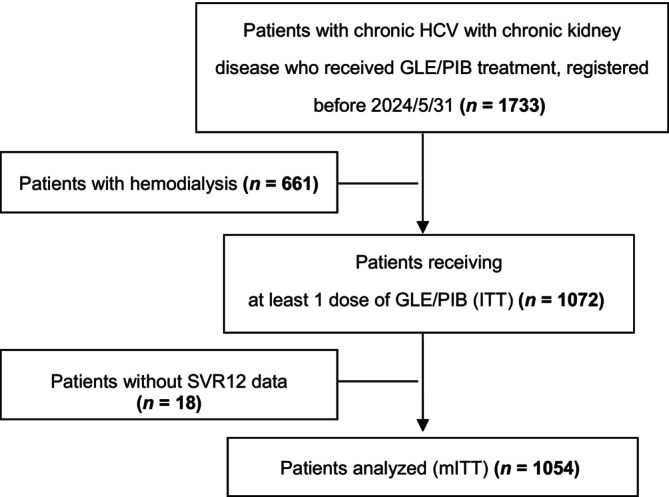
Flowchart of participant selection. DAA, direct‐acting antiviral; GLE/PIB, glecaprevir/pibrentasvir; ITT, intention‐to‐treat; mITT, modified intention‐to‐treat; SVR, sustained virologic response; SVR12, undetectable HCV RNA concentration throughout 12 weeks of posttreatment follow‐up.

Among the 1733 patients with chronic HCV with CKD who received GLE/PIB treatment, 661 on hemodialysis were excluded, along with 18 who lacked SVR12 data. Consequently, 1072 patients with available treatment outcomes were included in the ITT analysis (Figure 1). The mean age of these patients was 69.1 years, with men comprising 52.2% of the cohort. The predominant viral genotype was HCV genotype 2 (GT2), which was identified in 58.1% of the patients, followed by genotype 1 (GT1) in 28.3% of the patients. Additionally, 21 patients (2.0%) had genotype 3 (GT3) infection, and 74 (7.8%) had genotype 4/5/6 infection. At baseline, 189 patients (17.6%) had an HCV RNA concentration >6,000,000 IU/mL. Coinfections occurred in 63 patients (5.9%) with hepatitis B virus and 22 patients (2.1%) with HIV. The cohort also had common comorbidities: 579 patients (55.5%) had hypertension, 377 (35.7%) had diabetes, 338 (31.9%) had dyslipidemia, 49 (4.6%) had a history of cerebral vascular accidents, and 153 (14.3%) had cardiovascular disease. Additionally, 32 patients (3.0%) had a documented history of intravenous drug abuse. Compared with patients with early CKD, those with pre‐ESRD exhibited a higher prevalence of diabetes, dyslipidemia, cerebral vascular accidents, and GT2 infections. Moreover, patients with early CKD exhibited higher AST, ALT, albumin, and bilirubin levels; eGFRs; and platelet counts and lower FIB‐4 scores (Table 1).

**TABLE 1:** Baseline patient characteristics and clinical features.

Characteristics	Overall	Pre‐ESRD	Early CKD	*P* value
Mean ± SD or *N* (%)	(*N* = 1072)	(*N* = 584)	(*N* = 488)	(Pre‐ESRD vs. Early CKD)
Age (years), median (range)	69.1 ± 10.9	69.6 ± 11.3	68.5 ± 10.4	0.13
	70 (62–77)	70 (63–78)	69 (62–76)	
Age ≥65 years, *n* (%)	746 (69.6)	416 (71.2)	330 (67.6)	0.20
Male, *n* (%)	559 (52.2)	295 (50.5)	264 (54.1)	0.24
HCV genotype				
1a/1b, *n* (%)	330 (28.3)	168 (28.8)	135 (27.7)	0.03*
2, *n* (%)	623 (58.1)	354 (60.6)	269 (55.1)	
3, *n* (%)	21 (2.0)	7 (1.2)	14 (2.9)	
4/5/6, *n* (%)	84 (7.8)	36 (6.2)	48 (9.8)	
Mixed/unclassified, *n* (%)	41 (3.8)	19 (3.3)	22 (4.5)	
HCV RNA^a^, log_10_ IU/mL, median (range)	5.8 ± 1.2	5.8 ± 1.2	5.8 ± 1.1	0.77
	6.1 (5.1–6.6)	6.0 (5.0–6.6)	6.1 (5.2–6.6)	
HCV RNA^a^ >6,000,000 IU/mL, *n* (%)	189 (17.6)	109 (18.7)	80 (16.4)	0.32
AST, U/L	48.2 ± 42.0	45.0 ± 42.1	52.1 ± 41.7	0.01*
ALT, U/L	52.9 ± 54.5	47.2 ± 50.4	59.6 ± 58.3	<0.01*
Platelet count, ×10^3^ U/L	190.5 ± 72.1	187.3 ± 74.4	194.4 ± 69.2	0.11
Albumin, g/dL	4.1 ± 0.4	4.0 ± 0.4	4.2 ± 0.4	<0.01*
Total bilirubin, mg/dL	0.7 ± 0.3	0.6 ± 0.3	0.7 ± 0.3	<0.01*
Creatinine, mg/dL	2.3 ± 2.1	3.2 ± 2.6	1.3 ± 0.2	<0.01*
eGFR, mL/min/1.73 m^2^	39.1 ± 16.5	27.0 ± 12.8	53.5 ± 4.3	<0.01*
FIB‐4	3.0 ± 2.4	3.1 ± 2.8	2.8 ± 1.9	0.04*
Treatment naïve, *n* (%)	1012 (94.4)	558 (95.6)	454 (93.0)	0.07
Cirrhosis^b^, *n* (%)	171 (16.0)	100 (17.1)	71 (14.6)	0.25
Proteinuria (+)^a^, *n* (%)	172 (16.0)	121 (20.7)	51 (10.5)	<0.01*
Comorbidities, *n* (%)				
Hypertension^b^	579 (55.5)	319 (55.9)	260 (55.0)	0.77
Diabetes^b^	377 (35.7)	222 (38.5)	155 (32.3)	0.04*
Dyslipidemia^b^	338 (31.9)	200 (34.7)	138 (28.6)	0.03*
Cerebral vascular accident	49 (4.6)	19 (3.3)	30 (6.2)	0.02*
Cardiovascular diseases	153 (14.3)	81 (13.9)	72 (14.8)	0.68
HIV^a^, *n* (%)	22 (2.1)	4 (0.7)	18 (3.7)	<0.01*
PWID, *n* (%)	32 (3.0)	12 (2.1)	20 (4.1)	0.05
HBV^a^, *n* (%)	63 (5.9)	31 (5.3)	32 (6.6)	0.44

^a^ 1 patient without HCV RNA information, 732 patients without proteinuria information, 15 patients without diabetes information, 28 patients without hypertension information, 12 patients without dyslipidemia information, 119 patients without HIV information, and 63 patients without HBV information.

^b^ Liver cirrhosis: Self‐reported or FIB‐4 ≥6.5. Diabetes: Self‐reported with drug treatment or fasting glucose >126 mg/dL or postprandial glucose ≥200 mg/dL or HbA1c >6.5%. Dyslipidemia: HDL‐cholesterol ≤40 mg/dL (M) and ≤50 mg/dL (F) or triglycerides ≥150 mg/dL or self‐reported dyslipidemia with drug treatment. Hypertension: BP ≥130/85 mmHg or self‐reported HTN with drug treatment.

* *P* < 0.05.

### 3.2 Treatment responses

The overall SVR12 rates were 97.9% (1050/1072) in the ITT analysis and 99.6% (1050/1054) in the mITT analysis. When the results were stratified by kidney status, the SVR12 rates were 97.8% (571/584) and 99.7% (571/573) among patients with pre‐ESRD and 98.2% (479/488) and 99.6% (479/481) among those with early CKD, according to the ITT and mITT analyses, respectively. In the cohort, four patients experienced virological failure: three relapsed after treatment completion, whereas one had a virological breakthrough during the treatment period. Additionally, 18 patients experienced nonvirological failure due to loss of follow‐up (Table 2). A subgroup analysis conducted using the mITT approach revealed high SVR12 rates across all subgroups, irrespective of kidney status, viral genotype, comorbidities, and special population subgroup status. This high efficacy was also observed in patients with DAA compliance below 80%. Notably, in the mITT analysis, all six patients with adherence below 80% achieved a 100% SVR12 rate. However, in the ITT analysis, five patients with adherence below 80% did not have available SVR information (Table 3). Overall, up to 99% of patients maintained a drug adherence rate above 80% throughout the 8‐week regimen. This high adherence rate was consistent across both patients with pre‐ESRD (98.6%) and those with early CKD (99.4%; Table 4).

**TABLE 2:** Virologic responses (SVR12).

	Overall	Pre‐ESRD	Early CKD
	(*N* = 1072)	(*N* = 584)	(*N* = 488)
SVR12			
ITT, *n*/*N* (%)	1050/1072 (97.9)	571/584 (97.8)	479/488 (98.2)
mITT, *n*/*N* (%)	1050/1054 (99.6)	571/573 (99.7)	479/481 (99.6)
Reason for non‐SVR12 (*n* = 22)			
Virologic failure, *n*	4 (0.4)	2 (0.3)	2 (0.4)
Breakthrough, *n*	1 (0.1)	1 (0.2)	0 (0.0)
Relapse, *n*	3 (0.3)	1 (0.2)	2 (0.4)
Nonvirologic failure, *n*	18 (1.7)	11 (1.9)	7 (1.4)
Lost to follow‐up, *n*	18 (1.7)	11 (1.9)	7 (1.4)

**TABLE 3:** SVR12 (mITT) in subgroups, stratified by kidney status, determined using the Wilson score method.

Characteristics	Overall		Pre‐ESRD		Early CKD	
	(*N* = 1054)		(*N* = 573)		(*N* = 481)	
	*n*/*N* (%)	95% CI	*n*/*N* (%)	95% CI	*n*/*N* (%)	95% CI
HCV genotype						
1	293/295 (99.3)	97.6–99.8	164/164 (100.0)	97.7–100.0	129/131 (98.5)	94.6–99.6
2	612/614 (99.7)	98.8–99.9	345/347 (99.4)	97.9–99.8	267/267 (100.0)	98.6–100.0
3	21/21 (100.0)	84.5–100.0	7/7 (100.0)	64.6–100.0	14/14 (100.0)	78.5–100.0
4/5/6	83/83 (100.0)	95.6–100.0	36/36 (100.0)	90.4–100.0	47/47 (100.0)	92.4–100.0
Mixed/unclassified	41/41 (100.0)	91.4–100.0	19/19 (100.0)	83.2–100.0	22/22 (100.0)	85.1–100.0
Diabetes						
No	667/669 (99.7)	98.9–99.9	345/346 (99.7)	98.4–99.9	322/323 (99.7)	98.3–99.9
Yes	368/370 (99.5)	98.1–99.9	219/220 (99.5)	97.5–99.9	149/150 (99.3)	96.3–99.9
Cardiovascular disease						
No	900/904 (99.6)	98.9–99.8	493/495 (99.6)	98.5–99.9	407/409 (99.5)	98.2–99.9
Yes	150/150 (100.0)	97.5–100.0	78/78 (100.0)	95.3–100.0	72/72 (100.0)	94.9–100.0
Dyslipidemia						
No	710/712 (99.7)	99.0–99.9	369/370 (99.7)	98.5–99.9	341/342 (99.7)	98.3–99.9
Yes	328/330 (99.4)	97.8–99.8	195/196 (99.5)	97.2–99.9	133/134 (99.3)	95.9–99.9
Cerebral vascular accident						
No	1002/1006 (99.6)	99.0–99.8	552/554 (99.6)	98.7–99.9	450/452 (99.6)	98.4–99.9
Yes	48/48 (100.0)	92.6–100.0	19/19 (100.0)	83.2–100.0	29/29 (100.0)	88.3–100.0
PWID						
No	1019/1022 (99.7)	99.1–99.9	559/561 (99.6)	98.7–99.9	460/461 (99.8)	98.8–99.9
Yes	31/32 (96.9)	84.3–99.4	12/12 (100.0)	75.8–100.0	19/20 (95.0)	76.4–99.1
HIV^a^						
No	910/913 (99.7)	99.0–99.9	508/510 (99.6)	98.6–99.9	402/403 (99.8)	98.6–99.9
Yes	21/22 (95.5)	78.2–99.2	4/4 (100.0)	51.1–100.0	17/18 (94.4)	74.2–99.0
Adherence						
≥80%	1044/1048 (99.6)	99.0–99.9	568/570 (99.6)	98.7–99.9	476/478 (99.6)	98.5–99.9
<80%^b^	6/6 (100.0)	61.0–100.0	3/3 (100.0)	43.9–100.0	3/3 (100.0)	43.9–100.0

^a^ 119 patients without HIV information.

^b^ Adherence <80%: 5 patients without SVR information.

**TABLE 4:** Drug adherence.

	All patients	Pre‐ESRD	Early CKD
80%–100%, *n* (%)	1061 (99.0)	576 (98.6)	485 (99.4)
<80%, *n* (%)	11 (1.0)	8 (1.4)	3 (0.6)
60%–80%, *n* (%)	3 (0.3)	1 (0.2)	2 (0.4)
40%–60%, *n* (%)	4 (0.4)	3 (0.5)	1 (0.2)
20%–40%, *n* (%)	3 (0.3)	3 (0.5)	0 (0.0)
<20%, *n* (%)	1 (0.1)	1 (0.2)	0 (0.0)

### 3.3 Safety

As detailed in Table 5, 181 patients (16.8%) experienced AEs. The most common AEs, occurring in at least 1% of the patients, included nausea (1.4%), headache (2.3%), pruritus (8.6%), rash (1.7%), and insomnia (2.1%). SAEs were reported in nine patients (0.8%). Among these, one patient with severe itching and jaundice and another with dizziness were potentially related to DAA treatment. The TACR database includes proportions of patients with abnormal liver function; however, it does not typically record the causal relationships between AEs and laboratory abnormalities, which limited the current study's ability to conduct a comprehensive exploration of these connections. On average, patients had 4.9 ± 1.0 outpatient visits during GLE/PIB treatment (Table 6).

**TABLE 5:** Safety summary.

Event, *n* (%)	Overall	Pre‐ESRD	Early CKD
	(*N* = 1072)	(*N* = 584)	(*N* = 488)
Any adverse event	181 (16.8)	100 (17.1)	81 (16.6)
Serious adverse event	9 (0.8)	7 (1.2)	2 (0.4)
DAA‐related serious adverse event	2^a^ (0.2)	2 (0.3)	0 (0.0)
Discontinuation due to adverse event			
Temporary	4 (0.4)	3 (0.5)	1 (0.2)
Permanent	11 (1.0)	8 (1.4)	3 (0.6)
Adverse event occurring in ≥1% of patients			
Nausea	15 (1.4)	7 (1.2)	8 (1.6)
Headache	24 (2.3)	11 (1.9)	13 (2.7)
Pruritus	92 (8.6)	53 (9.1)	39 (8.0)
Rash	18 (1.7)	6 (1.0)	12 (2.5)
Insomnia	23 (2.1)	9 (1.5)	14 (2.9)
Total blood bilirubin increased^b^			
Grade 1	230 (21.5)	106 (18.2)	124 (25.4)
Grade 2	66 (6.2)	35 (6.0)	31 (6.4)
Grade 3–4	6 (0.6)	3 (0.5)	3 (0.6)
Grade 3–4 AST or ALT elevation	1 (0.1)	1 (0.2)	0 (0.0)
Alanine aminotransferase increased^c^			
Grade 1	58 (5.4)	42 (7.2)	16 (3.3)
Grade 2	4 (0.4)	3 (0.5)	1 (0.2)
Grade 3–4	4 (0.4)	2 (0.3)	2 (0.4)
Aspartate aminotransferase increased^c^			
Grade 1	49 (4.6)	33 (5.7)	16 (3.3)
Grade 2	5 (0.5)	2 (0.3)	3 (0.6)
Grade 3–4	4 (0.4)	3 (0.5)	1 (0.2)

^a^ DAA‐related serious adverse events: 1 patient reported severe itching and jaundice, 1 patient reported dizziness.

^b^ Total blood bilirubin increased: Grade1: 1.0 ULN − 1.5 × ULN if baseline was normal; >1.0 to 1.5 × baseline if baseline was abnormal. Grade2: >1.5 to 3.0 × ULN if baseline was normal; >1.5 to 3.0 × baseline if baseline was abnormal. Grade3: >3.0–10.0 × ULN if baseline was normal; >3.0–10.0 × baseline if baseline was abnormal. Grade 4: >10.0 × ULN if baseline was normal; >10.0 × baseline if baseline was abnormal.

^c^ Alanine aminotransferase/Aspartate aminotransferase increased: Grade1: >1.0 to 3.0 × ULN if baseline was normal; >1.5 to 3.0 × baseline if baseline was abnormal. Grade2: >3.0–5.0 × ULN if baseline was normal; >3.0–5.0 × baseline if baseline was abnormal. Grade3: >5.0–20.0 × ULN if baseline was normal; >5.0–20.0 × baseline if baseline was abnormal. Grade4: >20.0 × ULN if baseline was normal; >20.0 × baseline if baseline was abnormal. * *P* < 0.05.

**TABLE 6:** Health‐care resource utilization (number of clinic visits from DAA initiation to SVR12), determined using Wilcoxon rank sum test (Pre‐ESRD vs. Early CKD).

	All patients	Pre‐ESRD	Early CKD	*p* value*
Number of visits (BL, W1, W2, W3, W4, W6, W8/EOT, postM1, PostM3_6), mean ± SD (range)	4.86 ± 0.99	4.85 ± 0.98	4.88 ± 0.99	0.86
	5 (4–5)	5 (4–5)	5 (4–5)	

* *P* < 0.05.

### 3.4 Changes in laboratory data before and after patients achieved SVR12

A total of 1054 patients had laboratory data available at both baseline and at SVR12. Significant improvements were observed in liver‐related biochemical parameters, including ALT, AST, platelet counts, albumin levels, and FIB‐4 scores in both the overall patient population and across subgroups (Table 7). The eGFR demonstrated a significant increase from 39.2 ± 16.5 to 41.9 ± 21.8 mL/min/1.73 m^2^ (*p* < 0.01) overall. Notable improvements were observed in patients with early CKD, for whom the eGFR increased from 53.5 ± 4.3 to 57.1 ± 13.5 mL/min/1.73 m^2^ (*p* < 0.01), and in those with pre‐ESRD, for whom the eGFR increased from 27.1 ± 12.8 to 29.2 ± 19.1 mL/min/1.73 m^2^ (*p* < 0.01). However, no significant improvement in proteinuria was observed, which may be attributable to a substantial portion of data not being recorded in the TACR database.

**TABLE 7:** Changes in laboratory parameters from baseline to SVR12 in patients with chronic kidney disease using GLE/PIB, stratified by kidney status.

	Overall			Pre‐ESRD			Early CKD		
Mean ± SD	Baseline	SVR12	*p* value	Baseline	SVR12	*p* value	Baseline	SVR12	*p* value
AST, U/L	48.4 ± 42.2	24.7 ± 16.8	<0.01*	45.1 ± 42.2	23.9 ± 17.5	<0.01*	52.3 ± 41.8	25.7 ± 15.9	<0.01*
ALT, U/L	53.1 ± 54.6	19.8 ± 24.8	<0.01*	47.5 ± 50.6	18.5 ± 21.5	<0.01*	59.8 ± 58.4	21.4 ± 28.2	<0.01*
Total bilirubin, mg/dL	0.66 ± 0.31	0.66 ± 0.33	0.91	0.61 ± 0.31	0.61 ± 0.32	0.69	0.72 ± 0.30	0.72 ± 0.32	0.77
INR, sec	1.03 ± 0.31	1.01 ± 0.10	0.03*	1.03 ± 0.35	1.01 ± 0.11	0.18	1.03 ± 0.25	1.01 ± 0.08	0.06
Albumin, g/dL	4.1 ± 0.4	4.2 ± 0.4	<0.01*	4.0 ± 0.4	4.1 ± 0.4	<0.01*	4.2 ± 0.4	4.3 ± 0.4	<0.01*
Platelet, ×10^3^ U/L	188.6 ± 70.4	192.4 ± 68.5	0.02*	183.5 ± 69.6	187.7 ± 67.2	0.05	194.4 ± 71.0	197.7 ± 69.6	0.18
FIB‐4	3.0 ± 2.3	2.5 ± 1.6	<0.01*	3.2 ± 2.6	2.6 ± 1.5	<0.01*	2.8 ± 1.9	2.4 ± 1.7	<0.01*
Creatinine, mg/dL	2.3 ± 2.1	2.4 ± 2.3	0.13	3.2 ± 2.6	3.3 ± 2.7	0.06	1.3 ± 0.2	1.2 ± 0.3	0.01*
eGFR, mL/min/1.73^2^	39.2 ± 16.5	41.9 ± 21.8	<0.01*	27.1 ± 12.8	29.2 ± 19.1	<0.01*	53.5 ± 4.3	57.1 ± 13.5	<0.01*
Proteinuria^a^, *n*/*N* (%)	70/166 (42.2)	70/166 (42.2)	1.00	48/86 (55.8)	54/86 (62.8)	0.13	22/80 (27.5)	16/80 (20.0)	0.08

^a^ 732 patients without baseline proteinuria information, 874 patients without SVR12 proteinuria information.

* *P* < 0.05.

### 3.5 Association between comorbidities and changes in eGFR and proteinuria in patients with CKD before and after achieving SVR12

A total of 1054 patients were stratified on the basis of the presence of 1–4 of the following comorbidities: diabetes, hypertension, dyslipidemia, and cirrhosis. Changes in eGFR and proteinuria from baseline to SVR12 were analyzed. Among patients with CKD, those with a single comorbidity of hypertension demonstrated a significant change in eGFR, whereas those with a single comorbidity of dyslipidemia exhibited a borderline change in eGFR (Table 8). Additionally, all patients with CKD with 1–4 comorbidities exhibited significant changes in the proportion of proteinuria, with particularly notable changes observed in those with hypertension and dyslipidemia (Table 9).

**TABLE 8:** Changes in eGFR from baseline to SVR12 in patients with chronic kidney disease, stratified by four comorbidities of interest.

Diabetes	Hypertension	Dyslipidemia	Cirrhosis	*N*	Baseline eGFR	SVR12 eGFR	*p* value
Having 1 comorbidity							
Yes	No	No	No	33	41.9 ± 12.3	48.2 ± 22.7	0.07
No	Yes	No	No	145	42.8 ± 14.2	46.2 ± 19.3	<0.01*
No	No	Yes	No	29	44.8 ± 12.8	48.3 ± 16.8	0.06
No	No	No	Yes	42	37.8 ± 17.0	37.9 ± 18.9	0.92
Having 2 comorbidities							
Yes	Yes	No	No	102	39.2 ± 15.4	40.5 ± 19.2	0.21
Yes	No	Yes	No	22	39.5 ± 16.2	41.2 ± 21.8	0.53
Yes	No	No	Yes	9	36.3 ± 16.7	35.1 ± 22.6	0.74
No	Yes	Yes	No	85	40.7 ± 15.3	42.2 ± 19.4	0.24
No	Yes	No	Yes	21	40.0 ± 12.9	42.3 ± 16.5	0.40
No	No	Yes	Yes	6	50.0 ± 7.5	64.4 ± 20.6	0.06
Having 3 comorbidities							
Yes	Yes	Yes	No	135	37.6 ± 14.7	38.1 ± 19.4	0.61
Yes	Yes	No	Yes	35	41.4 ± 12.8	41.8 ± 16.9	0.80
Yes	No	Yes	Yes	6	37.5 ± 17.4	34.9 ± 20.7	0.51
No	Yes	Yes	Yes	18	41.4 ± 15.4	42.9 ± 15.4	0.55
Having 4 comorbidities							
Yes	Yes	Yes	Yes	31	36.5 ± 15.7	37.0 ± 18.5	0.69

* *P* < 0.05.

**TABLE 9:** Proportion of proteinuria changes from baseline to SVR12 in patients with chronic kidney disease, stratified by four comorbidities of interest.

Diabetes	Hypertension	Dyslipidemia	Cirrhosis	*N*	Baseline proteinuria (%)	SVR12 proteinuria (%)	*p* value
Having 1 comorbidity							
Yes	No	No	No	34	8 (23.5)	3 (8.8)	0.07
No	Yes	No	No	146	15 (10.3)	9 (6.2)	0.01*
No	No	Yes	No	30	6 (20.0)	4 (13.3)	0.03*
No	No	No	Yes	42	2 (4.8)	2 (4.8)	0.51
Having 2 comorbidities							
Yes	Yes	No	No	102	19 (18.6)	14 (13.7)	0.07
Yes	No	Yes	No	22	8 (36.4)	5 (22.7)	0.17
Yes	No	No	Yes	9	0 (0.0)	0 (0.0)	–
No	Yes	Yes	No	87	21 (24.1)	7 (8.1)	0.01*
No	Yes	No	Yes	22	2 (9.1)	1 (4.6)	0.72
No	No	Yes	Yes	7	0 (0.0)	1 (14.3)	0.80
Having 3 comorbidities							
Yes	Yes	Yes	No	137	44 (32.1)	26 (19.0)	<0.01*
Yes	Yes	No	Yes	36	9 (25.0)	1 (2.8)	0.03*
Yes	No	Yes	Yes	6	4 (66.7)	1 (16.7)	0.08
No	Yes	Yes	Yes	18	5 (27.8)	3 (16.7)	0.02*
Having 4 comorbidities							
Yes	Yes	Yes	Yes	31	25 (45.2)	6 (19.4)	0.01*

* *P* < 0.05.

## 4 DISCUSSION

This study represents the largest cohort of patients with CHC with early CKD or pre‐ESRD who were treated with an 8‐week GLE/PIB regimen. The study findings indicated high efficacy, with an SVR12 rate of 97.9% (1050/1072) in the ITT analysis. In the mITT population, after the exclusion of nonvirologic failures, the SVR12 rate was 99.6% (1050/1054). These results underscore that the GLE/PIB regimen is highly effective and well‐tolerated among patients with CHC with CKD in Taiwan. The high SVR12 rates were consistent across all HCV genotypes, comorbidities, and special population subgroups, including those with DAA adherence below 80% (Table 3). The findings further support the viability of simplifying HCV treatment with an 8‐week GLE/PIB regimen, particularly for nonspecialist care settings.^21^

In the mITT analysis, all six patients with adherence below 80% achieved a 100% SVR12 rate. However, the ITT analysis indicated that five patients with adherence below 80% lacked available SVR data (Table 3), which could have led to an overestimation of the SVR12 rate in the mITT analysis. Nevertheless, research has indicated that poor drug adherence is a predictor of treatment failure with DAAs.^19^, ^22^ Treatment of an extended duration, such as a 16‐week regimen of GLE/PIB, was reported to be associated with increased nonadherence.^22^ A pooled analysis of 10 clinical trials demonstrated a significant decline in drug adherence as treatment duration extended from 4 to 12 weeks.^23^ In a study in a real‐world setting involving 7203 patients who used injectable drugs, an 8‐week GLE/PIB regimen resulted in higher pill refill persistence than a 12‐week regimen did.^24^ A short‐course regimen should be selected to align with patient preferences^25^; such a regimen can improve adherence and facilitate decentralization of HCV care.^26^ In the present study, although the exact duration of GLE/PIB use was not recorded for patients with adherence below 80%, the high SVR12 rate suggests that near‐complete adherence is associated with successful outcomes.

Among the four patients who experienced virological failure, three had a relapse following treatment, and one had a virological breakthrough during treatment. Additionally, 18 patients were lost to follow‐up, resulting in nonvirological failure (Table 2). Factors commonly associated with DAA failure include previous treatment failures, resistance‐associated substitutions, decompensated liver cirrhosis, HCC, and poor adherence to medication.^27^ In a study by Lu et al., XGBoost machine learning algorithms were employed to effectively stratify the risk of DAA failure and identify key factors influencing such failure, such as body mass index, HCV RNA levels, platelet count, α‐fetoprotein, and the FIB‐4 index.^28^ Their method can be applied to facilitate the early identification of patients at risk for DAA treatment failure.

Among the reported AEs in this study, five—nausea, headache, pruritus, rash, and insomnia—occurred in at least 1% of the patient population, with pruritus being the most prevalent at 8.6%. Specifically, pruritus affected 9.1% of the patients with pre‐ESRD and 8.0% of those with early CKD. The registry revealed only one SAE, that is, one involving jaundice and pruritus, to be related to the DAA regimen. Although drug–drug interactions were not specifically examined in the registry, such interactions are generally manageable with appropriate pretreatment evaluations conducted by experienced health‐care providers. Research supports the safe use of GLE/PIB in patients with multiple comorbidities and complex medication regimens, including those with severe mental illness.^26^ In the present study, nine patients (0.8%) experienced SAEs, with one having a case of severe itching and jaundice and another experiencing dizziness potentially related to DAA use. Overall, the safety profile observed in this real‐world setting was deemed satisfactory.

Glecaprevir and pibrentasvir are not excreted through the kidneys, resulting in no significant increase in drug exposure for patients with kidney disease, thereby eliminating the need for dose adjustments.^29^ Consequently, GLE/PIB is recommended for patients with advanced CKD^9^, ^10^, ^11^ as well as those with diabetes.^12^ In the EXPEDITION‐5 study, the eGFR remained stable through posttreatment week 4 in a predialysis population.^8^ Research has also indicated that DAAs that lead to HCV eradication are beneficial to patients with CHC with CKD.^30^, ^31^ However, the effects of baseline renal function^30^ and sofosbuvir‐based therapy on changes in eGFR among patients treated with DAAs who achieve SVR^32^ remains a subject of debate. Variations in host baseline characteristics and discrepancies in posttherapy follow‐up durations may have contributed to these differing outcomes.^32^, ^33^ In our study, after SVR12 was achieved, the eGFR significantly increased from 39.2 ± 16.5 to 41.9 ± 21.8 mL/min/1.73 m^2^ (*p* < 0.01) overall. Notable improvements were observed in patients with early CKD, with the eGFR increasing from 53.5 ± 4.3 to 57.1 ± 13.5 mL/min/1.73 m^2^ (*p* < 0.01). In patients with pre‐ESRD, it increased from 27.1 ± 12.8 to 29.2 ± 19.1 mL/min/1.73 m^2^ (*p* < 0.01). Despite these improvements, no significant change in proteinuria was observed, which may be due to data being incomplete on the TACR database. A 10‐year cohort study in 2024,^34^ they indicated that renal function deterioration in SVR patients treated with IFN was typically transient, with eGFR levels returning to baseline over the long term (up to 10 years). By contrast, DAAs have demonstrated a long‐term adverse effect on eGFRs, with negative impacts potentially lasting up to 4.57 years. This suggests that reductions in the eGFR associated with DAA treatment may be either irreversible or require more than 4.57 years to normalize.

DAA therapy has been both qualitatively and quantitatively demonstrated to effectively resolve mixed cryoglobulinemia,^35^ which may lead to improvements in renal function.^36^ By addressing mixed cryoglobulinemia and mitigating its associated renal damage, DAA therapy can indirectly enhance longitudinal eGFR. Therefore, longer‐term follow‐up and ongoing monitoring of changes in eGFR and proteinuria changes are essential to fully assess the long‐term impact of the 8‐week GLE/PIB regimen on renal function.

Nearly two‐thirds of patients with CHC exhibit extrahepatic manifestations, such as non‐Hodgkin B‐cell lymphoma, mixed cryoglobulinemia, and an increased risk of metabolic abnormalities and cardiovascular events. These manifestations result from HCV infection of B lymphocytes and chronic inflammation.^4^, ^37^ Patients with CHC often exhibit impaired glucose tolerance and type 2 diabetes, independent of liver disease severity, and they are at a higher risk of cardiovascular morbidity and mortality, particularly when they have comorbid diabetes and hypertension.^38^ Additionally, cirrhosis in patients with CHC is associated with atherosclerosis, and abnormalities in lipid metabolism contribute to the progression of liver fibrosis.^39^ These comorbidities related to hepatitis C often interact with each other.

In our study, patients were categorized on the basis of the presence of 1–4 comorbidities, including diabetes, hypertension, dyslipidemia, and cirrhosis. Among the patients with CKD, those with hypertension demonstrated a significant change in eGFR, whereas those with dyslipidemia exhibited a borderline change (Table 8). All patients with CKD with comorbidities exhibited a significant change in proteinuria, with notable effects observed in those with hypertension and dyslipidemia (Table 9). Our previous study demonstrated that eradicating HCV can alleviate metabolic dysfunction‐associated steatotic liver disease (MASLD) in chronic hepatitis C (CHC) patients. Close monitoring of cardiometabolic risk factors is crucial for CHC patients with metabolic alterations, as these factors may change after HCV eradication and can be used to predict the progression of MASLD.^40^ The pathogenesis of HCV‐related extrahepatic manifestations remains incompletely understood. HCV may cause clonal B lymphocyte expansion, leading to immune complex deposition and subsequent vasculitis.^5^ Although theoretically, comorbidities associated with hepatitis C would be significantly correlated with improvements in eGFR and proteinuria, study limitations such as short observation periods or incomplete data may prevent researchers from obtaining robust findings.

This study has several limitations. Although the cohort size was large, the low prevalence of genotype 3, 4, and 5 HCV in Taiwan limited our ability to evaluate the real‐world efficacy of GLE/PIB in these populations. Moreover, because the data were derived from a registry rather than controlled clinical trials, AEs may have been underreported. We employed a standardized database platform to mitigate reporting bias and verify the results.

In conclusion, this study, representing the largest cohort of its kind from Taiwan, demonstrated that an 8‐week regimen of glecaprevir/pibrentasvir is both highly effective and well‐tolerated in treatment‐naïve patients with HCV with early CKD or pre‐ESRD. The therapy not only achieves high rates of SVR but also results in a significant improvement in renal function.

## CONFLICT OF INTEREST STATEMENT

Ming‐Lung Yu: research support (grant) from BMS, Gilead, Merck, and Roche diagnostics; consultant of AbbVie, BMS, Gilead, Roche, and Roche diagnostics; speaker of AbbVie, BMS, Eisai, Gilead, Roche and Roche diagnostics. Wan‐Long Chuang: advisory board member for AbbVie, BMS, Gilead, PharmaEssentia, Roche, and Vaccitech; speaker for AbbVie, BMS, Gilead, and Roche. Chia‐Yen Dai/Chung‐Feng Huang: Speaker for Abbvie, BMS, Gilead, Merck, and Roche. All other authors declare no competing interests.

## Data Availability

The data that support the findings of this study are available from the corresponding author upon reasonable request.

## References

[1] Polaris Observatory HCV Collaborators . Global change in hepatitis C virus prevalence and cascade of care between 2015 and 2020: a modelling study. Lancet Gastroenterol Hepatol. 2022;7(5):396–415. 35180382 10.1016/S2468-1253(21)00472-6

[2] Hepatitis C [Internet]. [cited 2024 Jul 28]. Available from: https://www.who.int/news-room/fact-sheets/detail/hepatitis-c

[3] Sagnelli E , Macera M , Russo A , Coppola N , Sagnelli C . Epidemiological and etiological variations in hepatocellular carcinoma. Infection. 2020;48(1):7–17. 31347138 10.1007/s15010-019-01345-y

[4] Cacoub P , Gragnani L , Comarmond C , Zignego AL . Extrahepatic manifestations of chronic hepatitis C virus infection. Dig Liver Dis. 2014;46:S165–S173. 25458776 10.1016/j.dld.2014.10.005

[5] Cacoub P , Saadoun D . Extrahepatic manifestations of chronic HCV infection. N Engl J Med. 2021;384(11):1038–1052. 33730456 10.1056/NEJMra2033539

[6] Fabrizi F , Cerutti R , Messa P . An updated view on the antiviral therapy of hepatitis C in chronic kidney disease. Pathogens. 2021;10(11):1381. 34832537 10.3390/pathogens10111381 PMC8619857

[7] Carrat F , Fontaine H , Dorival C , Simony M , Diallo A , Hezode C , et al. Clinical outcomes in patients with chronic hepatitis C after direct‐acting antiviral treatment: a prospective cohort study. Lancet. 2019;393(10179):1453–1464. 30765123 10.1016/S0140-6736(18)32111-1

[8] Lawitz E , Flisiak R , Abunimeh M , Sise ME , Park JY , Kaskas M , et al. Efficacy and safety of glecaprevir/pibrentasvir in renally impaired patients with chronic HCV infection. Liver Int. 2020;40(5):1032–1041. 31821716 10.1111/liv.14320

[9] Bhattacharya D , Aronsohn A , Price J , Lo Re V , AASLD‐IDSA HCV Guidance Panel. Hepatitis C Guidance . 2023 Update: American Association for the Study of Liver Diseases–Infectious Diseases Society of America recommendations for testing, managing, and treating hepatitis C virus infection. Clin Infect Dis. 2023. 10.1093/cid/ciad319 37229695

[10] Liver EA . For the S of the, chair: CPGP, Pawlotsky JM, et al. EASL recommendations on treatment of hepatitis C: final update of the series. J Hepatol. 2020;73(5):1170–1218. 32956768 10.1016/j.jhep.2020.08.018

[11] Martin P , Awan AA , Berenguer MC , Bruchfeld A , Fabrizi F , Goldberg DS , et al. Executive summary of the KDIGO 2022 clinical practice guideline for the prevention, diagnosis, evaluation, and treatment of hepatitis C in chronic kidney disease. Kidney Int. 2022;102(6):1228–1237. 36411019 10.1016/j.kint.2022.07.012

[12] Jang TY , Huang CF , Chang TS , Yang CC , Lo CC , Hung CH , et al. Impact of HCV eradication by directly acting antivirals on glycemic indices in chronic hepatitis C patients—a nationwide Taiwan HCV registry. J Formos Méd Assoc. 2024. 10.1016/j.jfma.2024.08.013 39168745

[13] Yang CC , Huang CF , Chang TS , Lo CC , Hung CH , Huang CW , et al. Real‐world efficacy and safety of universal 8‐week glecaprevir/pibrentasvir for treatment‐naïve patients from a nationwide HCV registry in Taiwan. Infect Dis Ther. 2024;13(6):1199–1213. 38679663 10.1007/s40121-024-00968-5 PMC11128429

[14] Huang CF , Kuo HT , Chang TS , Lo CC , Hung CH , Huang CW , et al. Nationwide registry of glecaprevir plus pibrentasvir in the treatment of HCV in Taiwan. Sci Rep. 2021;11(1):23473. 34873250 10.1038/s41598-021-03006-3 PMC8648748

[15] Chang TS , Huang CF , Kuo HT , Lo CC , Huang CW , Chong LW , et al. Effectiveness and safety of 8‐week glecaprevir/pibrentasvir in HCV treatment‐naïve patients with compensated cirrhosis: real‐world experience from Taiwan nationwide HCV registry. Hepatol Int. 2023;17(3):550–561. 36973633 10.1007/s12072-023-10506-z PMC10042416

[16] Huang CF , Tseng KC , Cheng PN , Hung CH , Lo CC , Peng CY , et al. Impact of sofosbuvir‐based direct‐acting antivirals on renal function in chronic hepatitis C patients with impaired renal function: a large cohort study from the nationwide HCV registry program (TACR). Clin Gastroenterol Hepatol. 2022;20(5):1151–1162.e6. 34333150 10.1016/j.cgh.2021.07.037

[17] Lo CC , Huang CF , Cheng PN , Tseng KC , Chen CY , Kuo HT , et al. Ledipasvir/sofosbuvir for HCV genotype 1, 2, 4–6 infection: real‐world evidence from a nationwide registry in Taiwan. J Formos Méd Assoc. 2022;121(8):1567–1578. 35123849 10.1016/j.jfma.2022.01.012

[18] Cheng PN , Mo LR , Chen CT , Chen CY , Huang CF , Kuo HT , et al. Sofosbuvir/velpatasvir for hepatitis C virus infection: real‐world effectiveness and safety from a nationwide registry in Taiwan. Infect Dis Ther. 2022;11(1):485–500. 34967920 10.1007/s40121-021-00576-7 PMC8847492

[19] Chen CY , Huang CF , Cheng PN , Tseng KC , Lo CC , Kuo HT , et al. Factors associated with treatment failure of direct‐acting antivirals for chronic hepatitis C: a real‐world nationwide hepatitis C virus registry programme in Taiwan. Liver Int. 2021;41:1265–1277. 10.1111/liv.14849 33655714 PMC8252422

[20] Lin MT , Hsu CN , Lee CT , Cheng SH . Effect of a pay‐for‐performance program on renal outcomes among patients with early‐stage chronic kidney disease in Taiwan. Int J Heal Polic Manag. 2021;0(8):1307–1315. 10.34172/ijhpm.2021.27 PMC9808322 33906336

[21] Yu ML , Tai CM , Mo LR , Kuo HT , Huang CF , Tseng KC , et al. An algorithm for simplified hepatitis C virus treatment with non‐specialist care based on nation‐wide data from Taiwan. Hepatol Int. 2024;18(2):461–475. 38246899 10.1007/s12072-023-10609-7 PMC11014878

[22] Brown A , Welzel TM , Conway B , Negro F , Bräu N , Grebely J , et al. Adherence to pan‐genotypic glecaprevir/pibrentasvir and efficacy in HCV‐infected patients: a pooled analysis of clinical trials. Liver Int. 2020;40(4):778–786. 31568620 10.1111/liv.14266 PMC7187170

[23] Zamor PJ , Brown A , Dylla DE , Dillon JF , Luetkemeyer AF , Feld JJ , et al. High sustained virologic response rates of glecaprevir/pibrentasvir in patients with dosing interruption or suboptimal adherence. Am J Gastroenterol. 2021;116(9):1896–1904. 34465693 10.14309/ajg.0000000000001332 PMC8389353

[24] Martinez A , Cheng WH , Marx SE , Manthena S , Dylla DE , Wilson L , et al. Shorter duration hepatitis C virus treatment is associated with better persistence to prescription refills in people who inject drugs: a real‐world study. Adv Ther. 2023;40(8):3465–3477. 37285080 10.1007/s12325-023-02539-5 PMC10329950

[25] Welzel TM , Yang M , Sajeev G , Chen YJ , Pinsky B , Bao Y , et al. Assessing patient preferences for treatment decisions for new direct acting antiviral (DAA) therapies for chronic hepatitis C virus infections. Adv Ther. 2019;36(9):2475–2486. 31240629 10.1007/s12325-019-01012-6 PMC6822851

[26] Huang CF , Jang TY , Yu SC , Huang SC , Ho SL , Yeh ML , et al. Patient‐centered and integrated outreach care for chronic hepatitis C patients with serious mental illness in Taiwan. Kaohsiung J Méd Sci. 2024;40(1):86–93. 37942784 10.1002/kjm2.12780 PMC11895585

[27] Huang CF , Yu ML . Unmet needs of chronic hepatitis C in the era of direct‐acting antiviral therapy. Clin Mol Hepatol. 2020;26(3):251–260. 32188235 10.3350/cmh.2020.0018 PMC7364348

[28] Lu MY , Huang CF , Hung CH , Tai CM , Mo LR , Kuo HT , et al. Artificial intelligence predicts direct‐acting antivirals failure among HCV patients: a nationwide HCV registry program. Clin Mol Hepatol. 2023;30(1):64–79. 38195113 10.3350/cmh.2023.0287 PMC10776298

[29] Kosloski MP , Dutta S , Zhao W , Pugatch D , Mensa F , Kort J , et al. THU‐230 pharmacokinetics, safety, and tolerability of next generation direct acting antivirals ABT‐493 and ABT‐530 in subjects with renal impairment. J Hepatol. 2016;64(2):S405–S406.

[30] D'Ambrosio R , Pasulo L , Giorgini A , Spinetti A , Messina E , Fanetti I , et al. Renal safety in 3264 HCV patients treated with DAA‐based regimens: results from a large Italian real‐life study. Dig Liver Dis. 2020;52(2):190–198. 31813755 10.1016/j.dld.2019.11.006

[31] Coppola N , Portunato F , Buonomo AR , Staiano L , Scotto R , Pinchera B , et al. Interferon‐free regimens improve kidney function in patients with chronic hepatitis C infection. J Nephrol. 2019;32(5):763–773. 30977055 10.1007/s40620-019-00608-z

[32] Tsai MC , Lin CY , Hung CH , Lu SN , Tung SY , Chien RN , et al. Evolution of renal function under direct‐acting antivirals treatment for chronic hepatitis C: a real‐world experience. J Viral Hepat. 2019;26(12):1404–1412. 31433885 10.1111/jvh.13193

[33] Álvarez‐Ossorio MJ , Sarmento E , Castro R , Granados R , Macías J , Morano‐Amado LE , et al. Impact of interferon‐free regimens on the glomerular filtration rate during treatment of chronic hepatitis C in a real‐life cohort. J Viral Hepat. 2018;25(6):699–706. 29377515 10.1111/jvh.12867

[34] Chang ML , Cheng JS , Chen WT , Hsu CW , Chen KH , Chen YC , et al. Long‐term renal function alterations in hepatitis C patients with SVRs: impacts of therapies and mixed cryoglobulinemia. J Infect Public Heal. 2024;17(3):486–494. 10.1016/j.jiph.2024.01.010 38280352

[35] Chang ML , Cheng JS , Chuang YH , Pao LH , Wu TS , Chen SC , et al. Evolution of Cryoglobulinemia in direct‐acting antiviral‐treated Asian hepatitis C patients with sustained virological responses: a 4‐year prospective cohort study. Front Immunol. 2022;13:823160. 35371039 10.3389/fimmu.2022.823160 PMC8964347

[36] Sise ME , Bloom AK , Wisocky J , Lin MV , Gustafson JL , Lundquist AL , et al. Treatment of hepatitis C virus‐associated mixed cryoglobulinemia with direct‐acting antiviral agents. Hepatology. 2016;63(2):408–417. 26474537 10.1002/hep.28297 PMC4718772

[37] Calogero A , Sagnelli E , Creta M , Angeletti S , Peluso G , Incollingo P , et al. Eradication of HCV infection with the direct‐acting antiviral therapy in renal allograft recipients. Biomed Res Int. 2019;2019:4674560. 31179323 10.1155/2019/4674560 PMC6507153

[38] Petta S , Maida M , Macaluso FS , Barbara M , Licata A , Craxì A , et al. Hepatitis C virus infection is associated with increased cardiovascular mortality: a meta‐analysis of observational studies. Gastroenterology. 2016;150(1):145–155.e4. 26386298 10.1053/j.gastro.2015.09.007

[39] Hsu WF , Lai HC , Chuang PH , Su WP , Chen SH , Chen HY , et al. Posttreatment nonalcoholic fatty liver disease fibrosis scores for predicting liver‐related complications in patients with chronic hepatitis C receiving direct‐acting antiviral agents. J Viral Hepat. 2022;29(9):785–794. 35657121 10.1111/jvh.13715

[40] Huang CF , Dai CY , Lin YH , Wang CW , Jang TY , Liang PC , et al. Dynamic change of MASLD in chronic hepatitis C patients after viral eradication: a nationwide registry study in Taiwan. Clin Mol Hepatol. 2024;30(4):883–894. 39069721 10.3350/cmh.2024.0414 PMC11540343

